# Utilizing SVD and VMD for Denoising Non-Stationary Signals of Roller Bearings

**DOI:** 10.3390/s22010195

**Published:** 2021-12-28

**Authors:** Qinghua Wang, Lijuan Wang, Hongtao Yu, Dong Wang, Asoke K. Nandi

**Affiliations:** 1 School of Mechatronic Engineering, Xi’an Technological University, Xi’an 710021, China; wqhhuazi@163.com (Q.W.); wlij_80@163.com (L.W.); yhtao1980@163.com (H.Y.); wangdong110104@163.com (D.W.); 2 Department of Electronic and Electrical Engineering, Brunel University London, London UB8 3PH, UK; 3 School of Mechanical Engineering, Xi’an Jiaotong University, Xi’an 710049, China

**Keywords:** singular value decomposition (SVD), variational mode decomposition (VMD), difference spectrum (DS) of singular value, roller bearing, denoising

## Abstract

In view of the fact that vibration signals of rolling bearings are much contaminated by noise in the early failure period, this paper presents a new denoising SVD-VMD method by combining singular value decomposition (SVD) and variational mode decomposition (VMD). SVD is used to determine the structure of the underlying model, which is referred to as signal and noise subspaces, and VMD is used to decompose the original signal into several band-limited modes. Then the effective components are selected from these modes to reconstruct the denoised signal according to the difference spectrum (DS) of singular values and kurtosis values. Simulated signals and experimental signals of roller bearing faults have been analyzed using this proposed method and compared with SVD-DS. The results demonstrate that the proposed method can effectively retain the useful signals and denoise the bearing signals in extremely noisy backgrounds.

## 1. Introduction

As a basic supporting component, rolling element bearings are widely used in rotating mechanical systems, and play an important role in a variety of industries. As a matter of fact, more than 50% of rotating machinery failures are related to bearing faults [1,2]. Therefore, it is very necessary for bearings to carry out condition monitoring and analysis, in which, early weak faults of the bearings can be detected, and the fault situation can be controlled in time. It will help to cut costs related to long downtimes, emergency maintenance and human injuries. Vibration signal analysis is widely used for the detection of bearing faults because of its easy measurement and high correlation with structural dynamics [3,4,5,6]. However, the main obstacle of bearing signal analysis from vibration data is that the collected non-stationary signals usually mixed with heavy noise caused by coupled machine components and the running environment [7,8]. It is difficult to extract effective features; nonetheless, several ideas on feature selection can be found in [9,10,11,12,13]. As it is difficult to detect early faults from the non-stationary signals with heavy and complex background noise, vibration signal denoising is an important step for early fault detection and diagnosis of bearing faults.

There are many signal decomposition methods proposed for signal denoising, such as wavelet transform (WT), empirical mode decomposition (EMD), variational mode decomposition (VMD), and so on. WT can deal with signals mixed with non-stationary noise signals by decomposing them into non-overlapping frequency bands, but its accuracy depends excessively on the selection of wavelet basis function and the decomposition scale [14,15]. EMD, an adaptive signal processing method, has been proposed for solving non-linear and non-stationary signal analysis by Hilbert Huang et al. in 1998 [16], which can adaptively decompose the signal into a series of intrinsic mode functions (Intrinsic Mode Function, IMF) from high frequency to low frequency according to the characteristic time scale of the signal itself. Although EMD makes up for the limitations of WT, it still has some shortcomings. For example, EMD will amplify the envelope estimation error due to multiple recursive decompositions, resulting in end effects, modal aliasing, and pseudo-pulse phenomena [17]. Variational mode decomposition (VMD) method is a non-recursive signal processing method with a firm theoretical foundation proposed by Dragomiretskiy and Zosso in 2014 [18]. It can not only overcome the mode aliasing in EMD but also obtain a better filtering effect based on its own Wiener filtering characteristics. Owing to the advantages of small end effect, high operation efficiency and good noise robustness, VMD has gained much attention by researchers since it was proposed [19,20,21,22]. However, the key step in the decomposition algorithm is to find the appropriate parameters K and α, where K is the number of intrinsic mode functions and α is the penalty factor, which affect the decomposition precision of IMFs [23]. Huang et al. [24,25] proposed an improved scale space guided VMD algorithm which included dividing resonance frequency bands of signal frequency band in scale space to determine the number of intrinsic modes in VMD, estimate the initial center frequency and corresponding penalty factor of each intrinsic function of VMD according to the boundary of resonance frequency band, and improve the adaptability and accuracy of VMD. These methods require a priori experience to determine key parameters through estimation. Some researchers use evolutionary algorithms (such as GA [26,27], PSO [28,29], etc.) to optimize parameters of VMD. Optimization methods with evolutionary algorithms can solve complex problems without a priori knowledge, but the results are not easy to interpret, and always consume a lot of computing time.

Singular value decomposition (SVD) is a powerful representation which can decompose a matrix into three component-matrices, exposing many of the useful and interesting properties of the original matrix. SVD can be used to denoise signals [30] as a kind of subspace algorithm. It can easily trade noise and signal quality by selectively removing singular values in the SVD pseudoinverse. However, it may cause serious distortion of a signal, especially when the signal contains some impact components. In order to solve the distortion of denoised signal of SVD and the dilemma of parameter selection of VMD, this paper proposes to combine the methods of SVD and VMD. Here, the parameter K is focused on optimization because the number of IMFs is more important, and has direct influence on exposing the components of signals. The main contributions of this paper are summarized as follows:

(1) SVD and difference spectrum (DS) of singular value are proposed to determine the number of intrinsic mode functions (IMFs) of VMD;
(2) The effective order of SVD and the kurtosis of VMD are used to select the IMFs of VMD, and non-stationary signals are denoised by reconstructing the selected components of VMD;
(3) The effectiveness and performance of the proposed method is verified using simulation bearing data and real experimental bearing data. These results are compared with those from SVD-DS.

The rest of this paper is arranged as follows. In Section 2 we introduce the theoretical basis of VMD, SVD, and difference spectrum (DS) of singular value. A novel denoising approach of combination of VMD and SVD is proposed in Section 3. The feasibility and performance of the proposed approach are discussed in Section 4 for simulated bearing signals and in Section 5 for real experimental bearing signals, respectively. Conclusions are drawn in Section 6.

## 2. Theoretical Basis

### 2.1. Singular Value Decomposition (SVD)

Singular value decomposition (SVD) has attracted a lot of attention because of its ability of noise filtering, disturbance in-sensitivity and high-resolution spectral factorization [31]. As pointed out by De Moor [32], SVD can also be applied to determine the structure of the underlying model which is contained in the singular values. The underlying model is referred to as signal and noise subspaces.

For a real matrix [*A*] of dimension *p* × *q*, there exists a *p* × *p* orthogonal matrix [*U*], a *q* × *q* orthogonal matrix [*V*] and a *p* × *q* diagonal matrix [Σ] (possibly a diagonal square matrix augmented with zero rows or columns), so that the following decomposition holds:

(1) $$A=U\Sigma V^{T}\;\Sigma =\left[\begin{array}{c} \sigma_{1}\\ \cdot \\ \cdot \\ \sigma_{p} \end{array}\right];\left(p=\min\left(m,n\right)\right)$$

where Σ contains the singular values ordered in descending order of magnitude, $\sigma_{1}>\sigma_{2}>\cdots >\sigma_{p}\geq 0$. *U* and *V* consist of the left and right singular vectors of *A*.

SVD reveals useful information about A. The number of non-zero singular values coincides with the rank *k* of *A*. if we choose a number r which is smaller than *k* and set (p−r) singular values to zero, we can construct a new rank-reduced singular matrix inversion.

(2) $$A^{\prime }=U\Sigma_{r}V^{T}$$

$A^{\prime }$ is an optimal lower-rank approximation to *A* and such a SVD is called truncated SVD. The value designed for r is very important because it will be a tradeoff between information loss and sensitivity to noise, which is called the number of effective singular values, and also called the effective order of the SVD.

### 2.2. Difference Spectrum of Singular Values

Difference spectrum of singular values [33] describes the changes of two adjacent singular values. There must be a peak in the difference spectrum which represents the boundary between useful signal and noise signal. It indicates that the singular value before and after the position has the largest difference in nature. Define the difference between adjacent singular values as follows:

(3) $$b_{i}=\sigma_{i}-\sigma_{i+1};\left(i=1,2,\cdots ,\left(p-1\right)\right)$$

Then, the sequence $B=\left[b_{1},b_{2},\cdots ,b_{p-1}\right]$ is called difference spectrum of singular values. The position of the maximum mutation point of the singular value can be found from the difference spectrum. The components corresponding to the r singular values before the mutation position are useful signals, and the components corresponding to other singular values after the mutation position are noise ones.

### 2.3. Variational Mode Decomposition (VMD)

VMD can effectively decompose non-stationary and nonlinear signals into a discrete set of quasi-orthogonal band limited intrinsic mode functions (IMFs) [18]. Each IMF component *u_k_* has a central frequency and a finite bandwidth. To evaluate the bandwidth of each mode, the corresponding constrained variational problem is given as follows:

(4) $$\left\{ \begin{array}{l} \underset{\{u_{k}\},\{\omega_{k}\}}{\min}{\{\sum_{k}\left\|\partial_{t}\left[\left(\delta (t)+\frac{j}{\pi t}\right)*u_{k}(t)\right]e^{j\omega_{k}t}\right\|}^{2}\\ s.t.\;\sum_{k}u_{k}=f \end{array}\right.$$

where, $\{u_{k}\}=\left\{u_{1},u_{2},\cdots ,u_{k}\right\}$ is a set of modal component functions, the sum of them is the original signal f. $\partial_{t}$ represents the gradient with respect to the time script t, $\{\omega_{k}\}=\left\{\omega_{1},\omega_{2},\cdots ,\omega_{k}\right\}$ is the center frequency set of the components, δ(t) denotes the impulse function, j is an imaginary unit, and ∗ represents the convolution operation.

To solve the above constrained problem, the quadratic penalty factor α and Lagrangian multiplier λ(t) are introduced to convert the constrained problem into the unconstrained variational problem. The augmented Lagrangian is expressed as

(5) $$\begin{array}{l} L\left(\left\{u_{k}\right\},\left\{\omega_{k}\right\},\lambda \right)=\alpha \sum_{k}{\left\|\partial_{t}\left[\left(\sigma (t)+\frac{j}{\pi t}\right)e^{-j\omega_{k}t}\right]\right\|}_{2}^{2}\\ +{\left\|f(t)-\sum_{k}^{K}u_{k}(t)\right\|}_{2}^{2}+\langle \lambda (t),f(t)-\sum_{k}^{K}u_{k}(t)\rangle \end{array}$$

In detail, the implementation process of the VMD is described as follows.

Step 1: Initialize mode $\left\{{\hat{u}}_{k}^{1}\right\}$, central frequency $\left\{{\hat{\omega }}_{k}^{1}\right\}$, Lagrangian multiplier $\lambda^{1}$, and iterations *n*.

Step 2: Execution cycle: *n* = *n* + 1.

Step 3: For all ω≥0, update $u_{k}$, $\omega_{k}$ and $\lambda_{k}$.

(6) $$u_{k}^{n+1}=\arg_{u_{k}}\min L(\{u_{i<k}^{n+1}\},\{u_{i\geq k}^{n}\},\{\omega_{i}^{n}\},\lambda^{n})$$

(7) $$\omega_{k}^{n+1}=\arg_{\omega_{k}}\min L(\{u_{i}^{n+1}\},\{\omega_{i<k}^{n+1}\},\{\omega_{i<k}^{n+1}\},\{\omega_{i\geq k}^{n}\},\lambda^{n})$$

(8) $$\lambda_{k}^{n+1}=\lambda^{n}+\tau (f-\sum_{k}u_{k}^{n+1})$$

Step 4: Repeat steps (2) to (3), until the convergence stop condition is satisfied. The stop condition is given as follows:

(9) $$\sum_{k}\left({\left\|u_{k}^{n+1}-u_{k}^{n}\right\|}_{2}^{2}/{\left\|u_{k}^{n}\right\|}_{2}^{2}\right)<\varepsilon$$

Step 5: Stop the iterations and obtain the IMF components.

More details of the VMD algorithm can be found in [18].

## 3. The Proposed SVD-VMD Methodology

**Figure 1:**
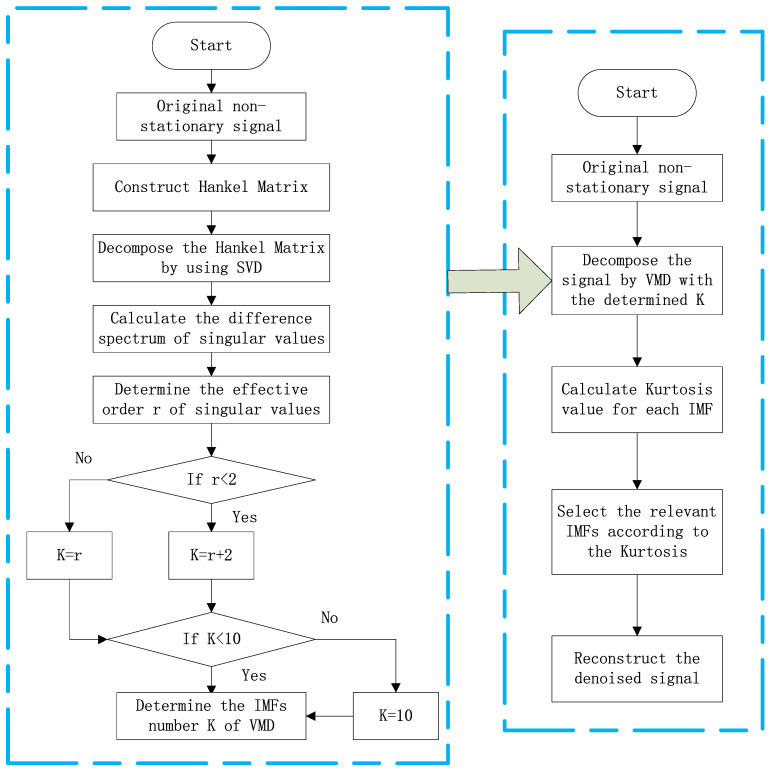
Flowchart of the proposed SVD-VMD denoising method.

Combining the above theoretical basis, a novel denoising method for non-stationary signals based on SVD-VMD algorithm is proposed. There are two stages in the algorithm. In the first stage, the effective singular value order is obtained which reveals the underlying model structure of the signals. The detailed process is shown in the left part of the Figure 1. At first, the vibration signal with one-dimension is converted into a matrix form. We construct Hankel matrix [34] to satisfy the above requirement. For a vibration signal X=[x(1),x(2),⋯,x(N)], a Hankel matrix can be formed as

$$H=\left[\begin{array}{c} x\left(1\right)\;x\left(2\right)\;\cdots \;x\left(n\right)\\ x\left(2\right)\;x\left(3\right)\;\cdots \;x\left(n+1\right)\\ \vdots \;\vdots \;\vdots \;\vdots \\ x\left(m\right)\;x\left(m+1\right)\;\cdots x\left(N\right) \end{array}\right]$$

where 1<m<N, n=N−m+1. Here, we set m=N/2.

Then, SVD is used to decompose the Hankel matrix and obtain two orthogonal matrices containing the left and right singular vectors, respectively, as well as a diagonal matrix containing the singular values ordered in descending order magnitude. Then, the difference spectrum of singular values is calculated, and the effective order r of singular values is determined according to the maximum mutation position of difference spectrum.

In the second stage, we use the obtained order to instruct VMD to decompose the original signal and select the relevant components to reconstruct denoised signal. The detailed process is shown in the right part of the Figure 1. Firstly, the original signal is decomposed by VMD with the number of IMFs K, where K is designed as (r + 2) when r is smaller than 3, else K is equal to r. When k is greater than 10, we set k as 10. The IMFs are obtained by decomposing the original signal with VMD, and the kurtosis values are calculated for each IMF. Finally, the relevant component corresponding to the maximum kurtosis value is selected to reconstruct the denoised signal.

## 4. Simulative Case Study

**Figure 2:**
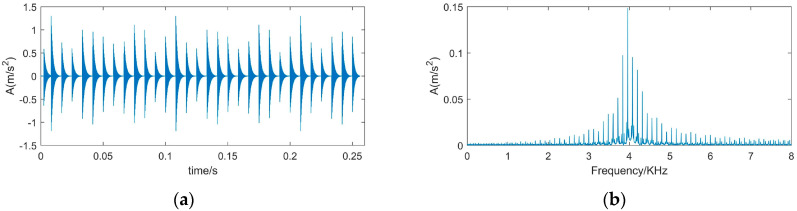
Time-domain waveform and frequency spectrum of impulsive signal. (**a**) Waveform of impulsive signal; (**b**) Spectrum of impulsive signal.

**Figure 3:**
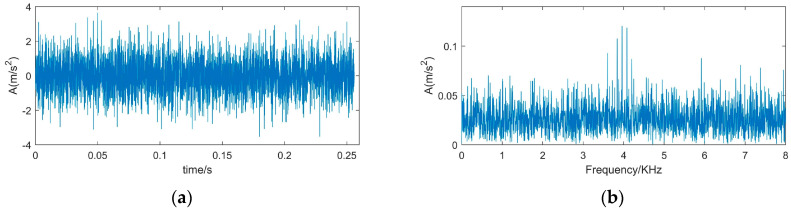
Time-domain waveform and frequency spectrum of simulated signal. (**a**) Waveform of simulated signal (**b**) spectrum of simulated signal.

To verify the effectiveness of the proposed method, we used a simulated signal [35] which simulates the impact signal generated by the inner ring fault, and the signal was contaminated by white Gaussian noise. The time diagram and the spectrum of the impulsive signal are shown in Figure 2, and the time-domain waveform and frequency spectrum of the simulated signal are shown in Figure 3. The description of the simulation signal is shown in Equation (10).

(10) $$\{\begin{array}{c} x\left(t\right)=s\left(t\right)+n\left(t\right)=\sum_{i}A_{i}h\left(t-iT\right)+n\left(t\right)\\ h\left(t\right)=\exp\left(-Ct\right)\cos\left(2\pi f_{n}t\right)\\ A_{i}=1+A_{0}\cos\left(2\pi f_{r}t\right) \end{array}$$

in which, x(t) represents is the observed signal, s(t) simulates the periodic impact component, and *n*(*t*) is the Gaussian noise signal, where SNR is −13 dB. $A_{0}$ is the amplitude which is set to be 0.3 and $f_{r}$ is the rotation frequency which is 30 Hz. *C* is the attenuation coefficient of 700 and resonance frequency is set to 4 KHz. The characteristic frequency of inner ring failure is 120 Hz. sampling frequency is 12 KHz and the signal length is 4096.

When the impulsive signal is weak or it is covered by strong background noise, the impulsive frequency may be hidden by noise. The simulated signal is formed by adding the Gaussian noise signal with SNR −13 dB to the impulsive signal. Figure 3 is the time-domain waveform and the frequency spectrum of the simulated signal.

Comparing Figure 3 with Figure 2, it is found that the periodic impact components in the simulated signal are completely submerged by noise, the impulsive frequency is hidden by noise, and the resonance frequency is also faintly visible.

**Figure 4:**
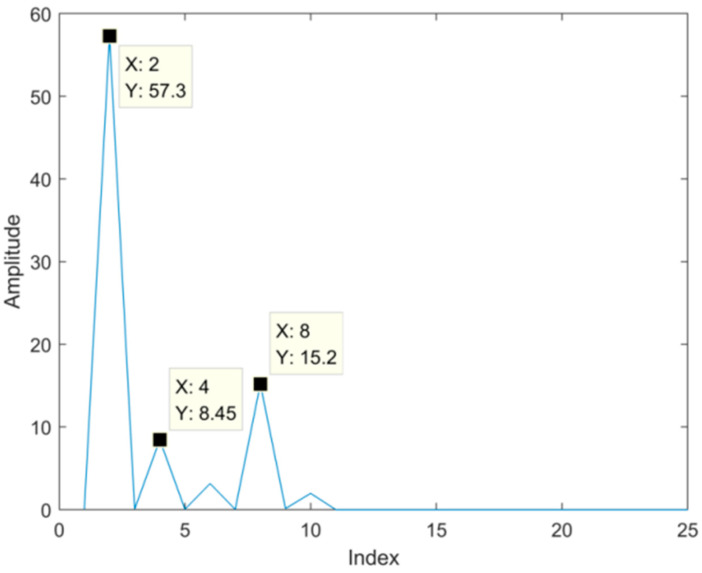
Difference spectrum of singular values.

Now we use the proposed SVD-VMD method to denoise the simulated signal. Firstly, we convert the simulated signal into the Hankel matrix and use SVD to decompose the Hankel matrix. The difference spectrum of singular values is shown in Figure 4. There are three peaks on the difference spectrum and the maximum peak occurs at the second index which has a far bigger peak than the other two peaks. It indicates that the properties of singular values are quite different before and after the position of the maximum peak. Therefore, we determine the effective order r of singular values to be two.

**Figure 5:**
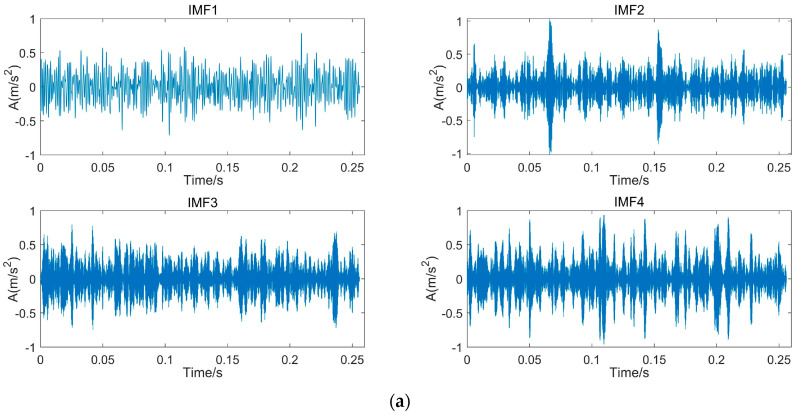

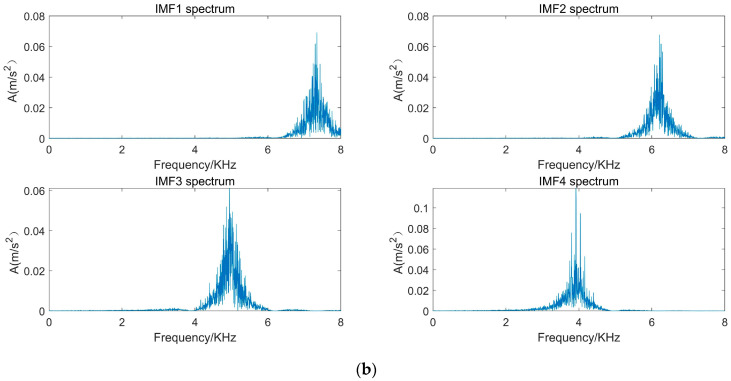
Decomposition result of VMD for the simulated signal. (**a**) Time domain decomposition result; (**b**) Frequency domain decomposition result.

According to the above description of the SVD-VMD method, the IMFs number of VMD is set to be four according to the effective order r of singular values. The decomposition result of VMD for the simulated signal is shown in Figure 5. From the time-domain waveform in Figure 5, the second and the fourth IMFs have obvious impact components.

**Figure 6:**
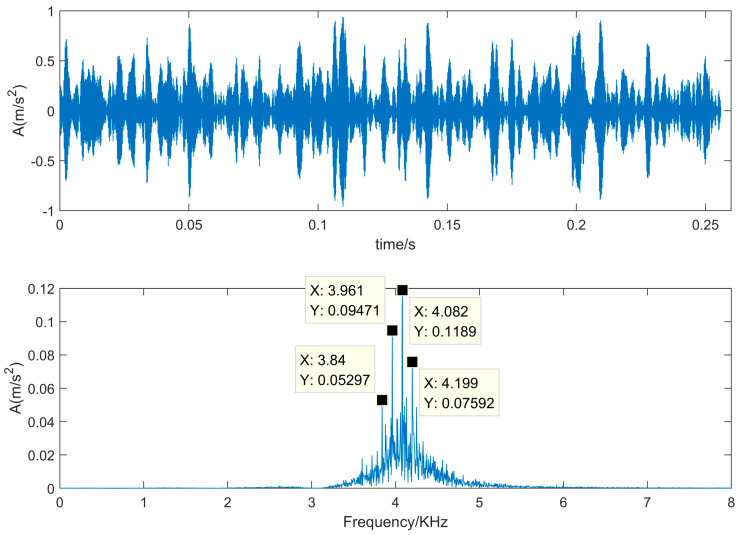
Denoised signal by using SVD-VMD algorithm.

In order to select the component with the useful information, the kurtosis value is calculated for each IMF, which are recorded in Table 1. In Table 1, the fourth component has the maximum kurtosis value. Therefore, the fourth component is selected to reconstruct the denoised signal. Figure 6 is the denoised signal by using the proposed SVD-VMD algorithm.

In Figure 6, the time domain waveform of the denoised signal reveals the obvious impulsive components. Furthermore, from the frequency spectrum of the denoised signal, it not only exposes that the resonance frequency is 4 KHz corresponding to the maximum peak, but also exposes that the impulsive frequency is 0.12 KHz corresponding to the frequency difference of the adjacent peaks.

**Figure 7:**
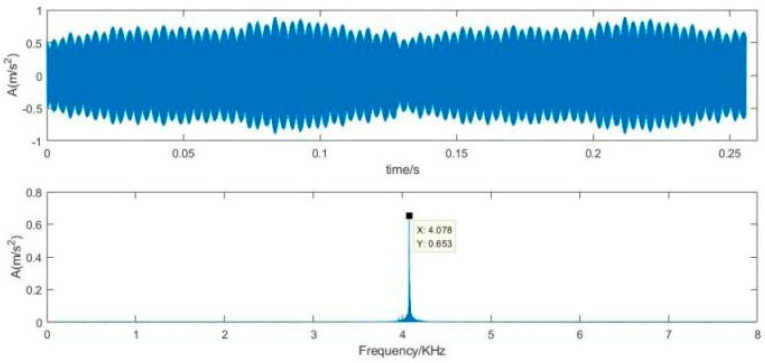
Denoised signal by reconstructing components of SVD-DS.

As we know, SVD itself can also be used for noise reduction if the effective order of singular values is found. The components corresponding to the effective order are useful and the other components are noise. There are many methods to determine the effective order of singular values such as the difference spectrum of singular values, singular value mean and singular value median. In this paper, we use difference spectrum (DS) of singular values to determine the effective order. This denoising method is called SVD-DS method. The denoised result is achieved by using SVD-DS is shown in Figure 7. We can see that the reconstructed signal is distorted and the impulsive components are nearly submerged in the noise due to its small intensity. From the frequency spectrum of the reconstructed signal, only resonance frequency can be seen, and the impulsive frequency has completely disappeared.

In order to evaluate quantitatively the noise reduction performance of the denoising methods, two indicators, namely, the output signal-to-noise ratio ($SNR_{out}$) and the root-mean-square error (RMSE) were used. The definition of $SNR_{out}$ and RMSE are described as follows:

$$SNR_{out}=10log\frac{\sum_{n=1}^{N}f^{2}\left(n\right)}{\sum_{n=1}^{N}{\left[f^{\prime }\left(n\right)-f\left(n\right)\right]}^{2}}$$

$$RMSE=\sqrt{\frac{1}{N}\sum_{n=1}^{N}{\left[f^{\prime }\left(n\right)-f\left(n\right)\right]}^{2}}$$

where, $f^{\prime }\left(n\right)$ is the denoised signal and f(n) is the original signal, and N is the length of the signal.

Table 2 describes the comparison between SVD-DS and SVD-VMD in terms of SNR and RMSE. The denoised signal by using SVD-VMD has higher signal-to-noise ratio and a smaller RMSE than that by using SVD-DS.

The denoising performance of *SNR_out_* and *RMSE* by the abovementioned methods for the noisy simulated non-stationary signal with different $SNR_{in}$ from −20 dB to 5 dB is displayed in Table 3.

In Table 3, the proposed SVD-VMD method can denoise the signals with improved $SNR_{out}$ at different $SNR_{in}$ but SVD-DS method can obtain the improved $SNR_{out}$ only at $SNR_{in}-20$ dB. Compared with SVD-DS method, the proposed SVD-VMD method showed the better performance of $SNR_{out}$ and RMSE at all different $SNR_{in}$.

## 5. Experimental Case Study

**Figure 8:**
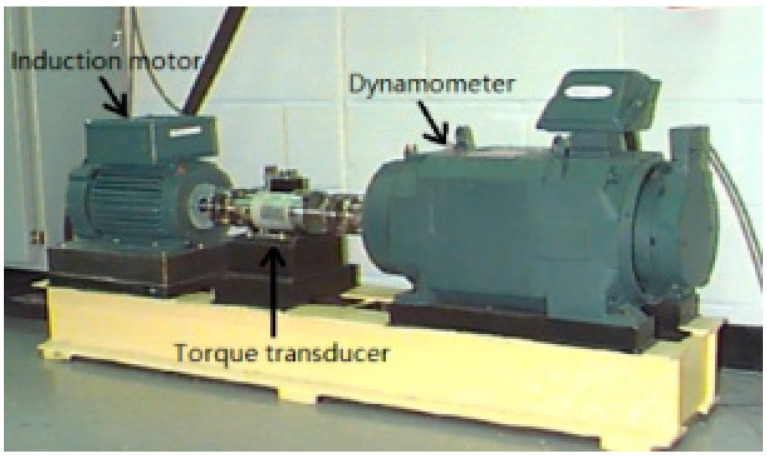
Roller bearing test rig [36].

**Figure 9:**
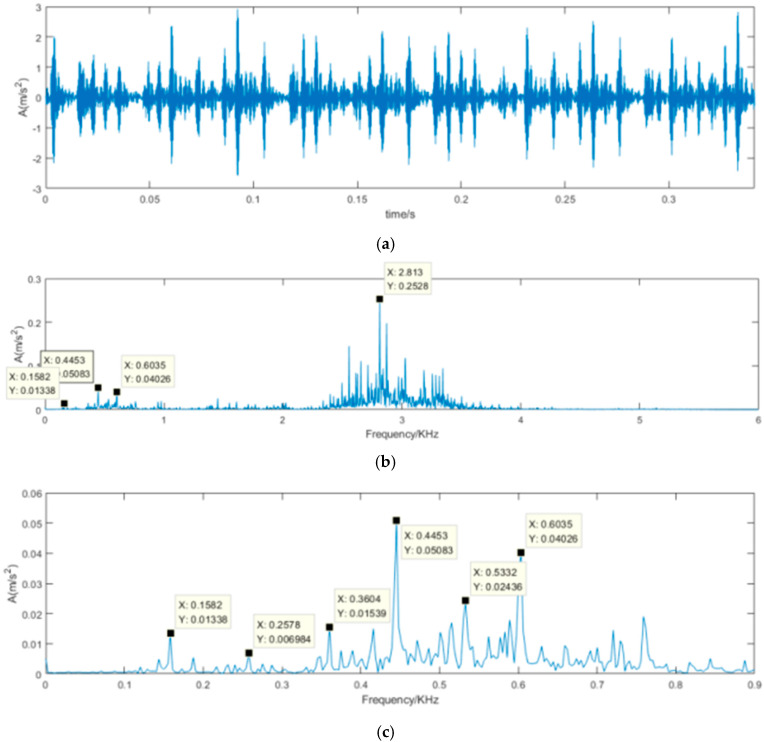
Time-domain waveform and frequency spectrum of vibration signal with inner ring failure. (**a**) Time-domain waveform of vibration signal with inner ring failure; (**b**) Frequency spectrum of vibration signal with inner ring failure; (**c**) Frequency spectrum limited in 0.9 KHz of vibration signal with inner ring failure.

The real roller bearing vibration signal data are used to verify further the effectiveness of the proposed SVD-VMD method; these experimental data come from the electrical engineering laboratory of Case Western Reserve University [36]. The test bearings are drive-end bearings (6205-2RSJEM SKF, deep groove ball bearing), which support the motor shaft. As shown in Figure 8, a 2-hp motor is set on the left of test bed, and a torque transducer and encoder are located at the center of the test bed, and a dynamometer is arranged on the right of the test bed. The dynamometer is controlled so that desired torque load levels can be achieved. Single point faults were introduced to the test bearings using electrical-discharge machining with fault diameters of 21 mil, (1 mil = 0.001 inches). A 16-channel DAT recorder is used to collect vibration signals with a sampling frequency of 12 kHz per channel. Each data set contains 1.2 × 10^5^ points. The experimental rotating frequency is about 30 Hz. The vibration signal of the rolling bearing with the inner ring failure is shown in Figure 9. The calculated defect frequency is 5.4152 times the shaft rotational speed (Hz). Since the shaft rotational speed is 1797 rpm (corresponding to the rotational frequency fr = 29.2 Hz), the inner ring fault frequency is 162.19 Hz.

From Figure 9, we can see that the maximum frequency spectrum is focused in 2.5~3.5 KHz of the frequency band. In order to observe the inner fault frequency, the frequency spectrum limited in 0.9 KHz is shown in Figure 9c. The inner ring fault frequency is 158.2 Hz which is close to the calculated 162.19 Hz, we also clearly observe 257.8 Hz (158.2 + 3*fr), 360.4 Hz (158.2 + 6*fr), 445.3 Hz (158.2 + 9*fr), and so on. These frequency components are their harmonics.

**Figure 10:**
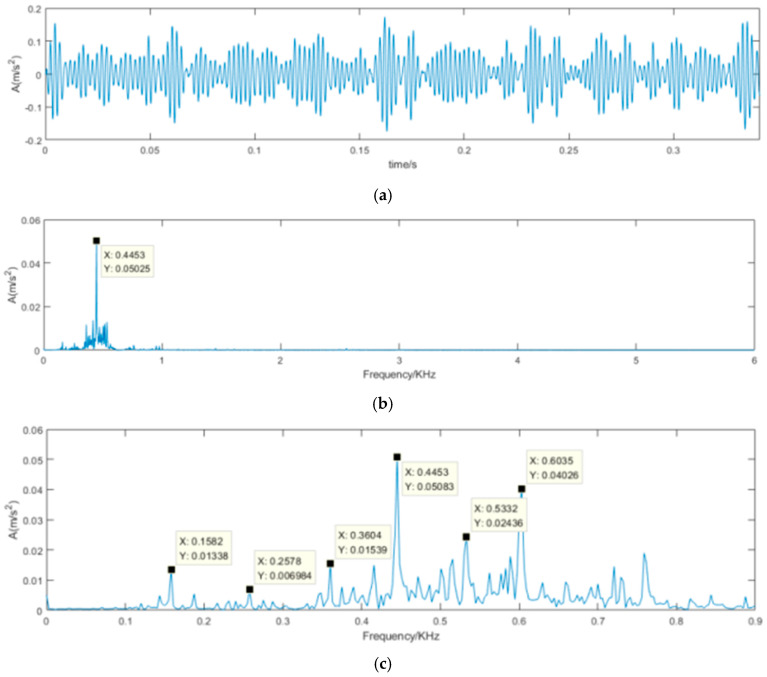
Time-domain waveform and frequency spectrum of denoised signal using SVD-VMD. (**a**) Time-domain waveform of denoised signal using SVD-VMD; (**b**) Frequency spectrum of denoised signal using SVD-VMD; (**c**) Frequency spectrum limited in 0.9 KHz of denoised signal using SVD-VMD.

**Figure 11:**
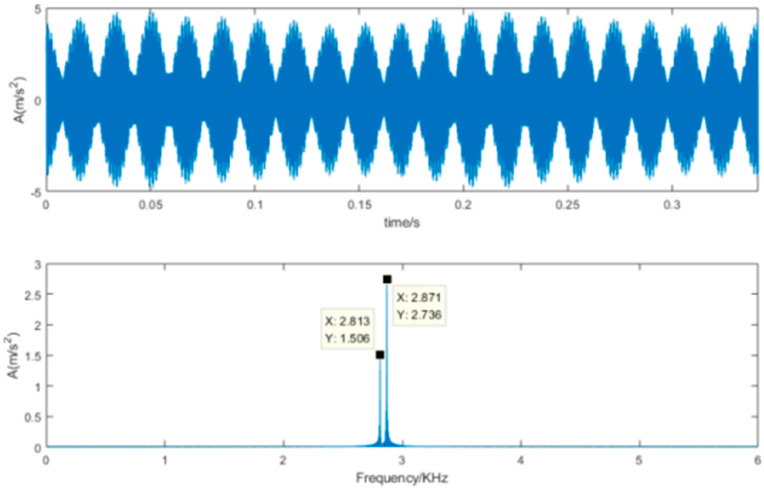
Time-domain waveform and frequency spectrum of denoised signal using SVD-DS.

The denoising results of the proposed SVD-VMD method is shown Figure 10. Compared Figure 10b with Figure 9b, the noise signal with high frequency has been diminished and the useful signal containing the inner ring fault has been kept and easy to identify. Figure 11 is the results of the SVD-DS method. It can reveal the periodic impulsive signal but cannot identify the inner ring fault frequency.

**Figure 12:**
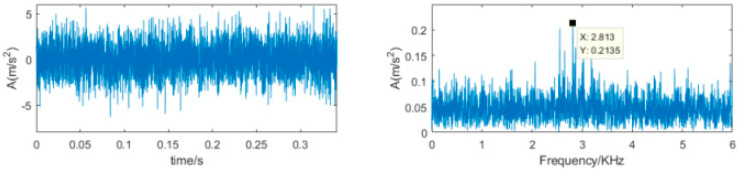
Time-domain waveform and frequency spectrum of the vibration signal with strong noise.

The collected vibration signal in Figure 9 has clear properties because there are obvious impulsive components from the time-domain waveform of Figure 9a. In order to verify the capability of the proposed method for dealing with the non-stationary signals containing strong noise, Gaussian noise signal with −13 dB of SNR has been added to the collected bearing signal, which is shown in Figure 12. The time-domain waveform has not obvious impulsive components which is submerged in the strong noise and the frequency spectrum distributed in the whole frequency band and has not obvious characteristics.

**Figure 13:**
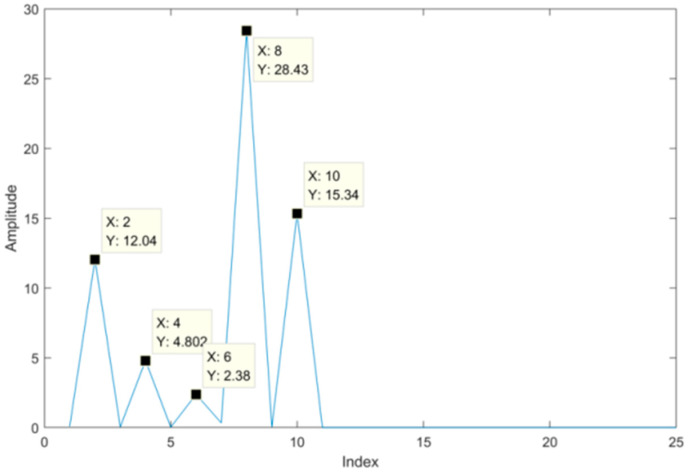
Difference spectrum of singular values for the bearing signal with strong noise.

To use the proposed SVD-VMD method, we construct Hankel matrix and decompose the matrix by SVD, then calculate the difference spectrum of singular which is shown in Figure 13. According to the difference spectrum, we find the maximum mutation position, from which we can determine the effective order of singular values. In Figure 13, there are four peaks and the maximum peak is at the index of eight which is larger than the other peaks.

**Figure 14:**
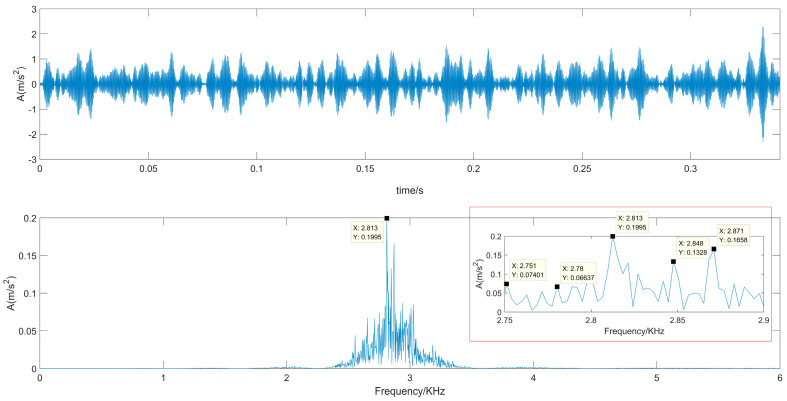
Time-domain waveform and frequency spectrum of the denoised signal using SVD-VMD.

According to the effective order, we decompose the vibration signal with strong noise into eight intrinsic mode functions. The kurtosis of each mode is calculated in Table 4. The kurtosis value at the sixth mode has the maximum value which is far larger than the other modes. The sixth component is selected to reconstruct denoised signal which is described in Figure 14. Compared the time-domain waveform of Figure 14 with that of Figure 12, it is visible that the denoised signal can reveals obvious impulsive components. Due to interference of the noise intensity, the components of inner ring fault frequency which are always low frequency are diminished as the noise ones. From the retained spectrum we can get 30 Hz of the rotational frequency.

**Figure 15:**
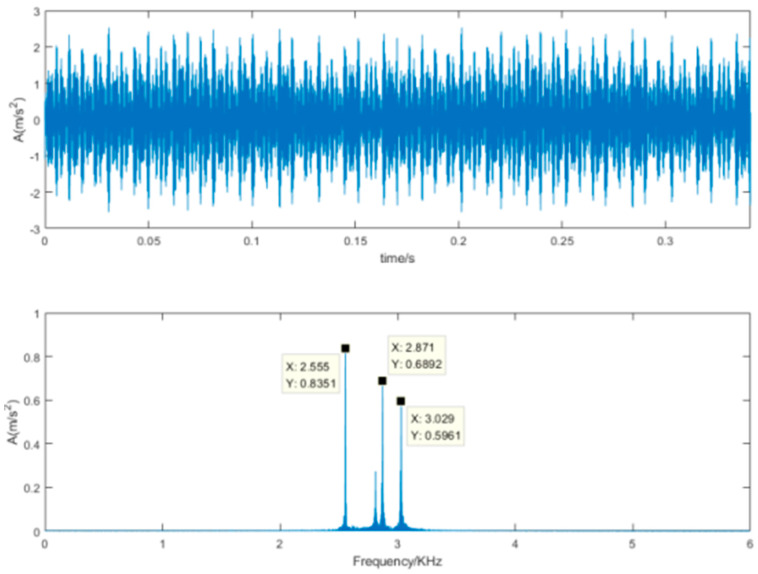
Time-domain waveform and frequency spectrum of the denoised signal using SVD-DS.

We also reconstructed the components corresponding to the effective order of singular values. The denoised results are shown in Figure 15. The time-domain waveform reveals that the signal contains impulsive components and the frequency spectrum is also focused on the high frequency. Although the frequency spectrum is very clear, we cannot get any fault frequency or rotational frequency. Comparing the frequency spectrum of Figure 14 and Figure 15 with that of Figure 13, the peak of Figure 14 is very close to that of Figure 13, but the peak of Figure 15 is very different, whatever the peak value or the corresponding frequency. In order to quantify the evaluation of denoising effectivity, the SNR and RMSE are calculated for the two methods in Table 5. It should be noted that the collected vibration signal is treated as the clear signal for the convenience of calculation. It is obvious that the proposed SVD-VMD has better performance even for the real experimental signal with strong noise.

## 6. Conclusions

This paper has proposed a novel denoising method which utilizes both SVD and VMD. SVD is used to expose the underlying model of the signal, where we obtain the effective order of singular values by calculating the difference spectrum of singular values. The obtained effective order guides VMD to set the appropriate numbers of IMFs. Calculating the kurtosis value for each IMF, the IMF corresponding to the maximum value is selected to reconstruct the denoised signal. Compared with the evolutionary algorithm, this method is not a black box, and the results can be explained. Moreover, it does not require a lot of computation and time. Compared with SVD-DS, the proposed method has superior denoising performance when it is applied to the simulation signals and the real experimental signals of the roller bearing faults. The results demonstrate that, whether it is for high signal-to-noise ratio signals or low signal-to-noise ratio signals, the proposed SVD-VMD performs better.

It should be noted that there are other denoising techniques, e.g., K-SVD and compressed sensing, which have not been studied here. We consider the proposed denoising technique in this paper will be effective for vibration signals from rotating machines. We also anticipate that this technique will work with some other types of signals. In the future, we may use this new denoising method in the acoustic analysis and compare the advantages/disadvantages between vibration analysis and acoustic analysis.

## Figures and Tables

**Table 1:** Kurtosis values of IMFs for simulated signals.

IMF	1	2	3	4
Kurtosis value	29.06	31.18	24.52	71.65

**Table 2:** Comparison between SVD-DS and SVD-VMD for simulated signals.

	SVD-DS	SVD-VMD
*SNR_out_* (dB)	−7.16	−0.65
*RMSE*	0.49	0.23

**Table 3:** Denoising performance with different *SNR_in_* (dB).

*SNR_in_*	*SNR_out_* of SVD-DS	*SNR_out_* of SVD-VMD	*RMSE* of SVD-DS	*RMSE* of SVD-VMD
−20	−18.09	−7.66	1.72	0.52
−10	−17.57	1.57	1.62	0.18
−5	−17.31	4.19	1.57	0.13
−1	−15.44	4.24	1.27	0.13
5	−14.52	3.41	1.14	0.14

**Table 4:** Kurtosis values of IMFs for experimental signals.

IMF	1	2	3	4	5	6	7	8
Kurtosis value	34.05	31.67	32.41	34.75	20.76	50.10	26.04	30.88

**Table 5:** Comparison between SVD-DS and SVD-VMD for experimental signals.

	SVD-DS	SVD-VMD
*SNR_out_* (dB)	−6.58	1.60
*RMSE*	1.10	0.43

## Data Availability

Not applicable.
